# Coenzyme Q10 and Endocrine Disorders: An Overview

**DOI:** 10.3390/antiox12020514

**Published:** 2023-02-17

**Authors:** David Mantle, Iain Parry Hargreaves

**Affiliations:** 1 Pharma Nord (UK) Ltd., Morpeth NE61 2DB, UK; 2 School of Pharmacy and Biomolecular Sciences, Liverpool John Moores University, Merseyside L3 5UX, UK

**Keywords:** coenzyme Q10, hyperthyroidism, diabetes, male infertility, polycystic ovary syndrome

## Abstract

Mitochondrial dysfunction and oxidative stress have been implicated in the pathogenesis of a number of endocrine disorders; this, in turn, suggests a potential role for the vitamin-like substance coenzyme Q10 (CoQ10) in the pathogenesis and treatment of these disorders, on the basis of its key roles in mitochondrial function, and as an antioxidant. In this article we have therefore reviewed the role of CoQ10 deficiency and supplementation in disorders of the thyroid, pancreas, gonads, pituitary and adrenals, with a particular focus on hyperthyroidism, type II diabetes, male infertility and polycystic ovary syndrome.

## 1. Introduction

Mitochondrial dysfunction and oxidative stress have been implicated in the pathogenesis of a number of endocrine disorders, including those of the thyroid, pancreas, gonads, pituitary and adrenals, as detailed in the following sections of this article. This in turn suggests a potential role for the vitamin-like substance coenzyme Q10 (CoQ10) in the pathogenesis and treatment of these disorders, on the basis of its key roles in mitochondrial function, and as an antioxidant [1]. In addition to its role as an electron carrier in the mitochondrial respiratory chain during ATP synthesis, CoQ10 in reduced (ubiquinol) form is a key antioxidant. CoQ10 is present in both the cellular and intracellular membranes (e.g., mitochondria, lysosomes, peroxisomes, endoplasmic reticulum), protecting the membranes from free radical induced oxidative damage. CoQ10 is the only endogenously synthesised lipid soluble antioxidant. CoQ10 provides antioxidant protection either by reaction with free radicals directly, or by regenerating the antioxidants vitamin C and vitamin E respectively. The oxidised and reduced forms of CoQ10, ubiquinone and ubiquinol, are continuously interconverted as part of the normal functioning of CoQ10; once oxidised ubiquinone is converted back to ubiquinol via the action of a number of oxidoreductase enzymes such as thioredoxion reductase. CoQ10 in ubiquinol form is bound to, and responsible for the antioxidant protection of, circulatory lipoproteins (LDL-, VLDL-, HDL-cholesterol) [1]. In this article we have therefore reviewed the role of CoQ10 deficiency and supplementation in Graves’ disease, Hashimoto’s disease, diabetes, infertility, Cushing’s disease, Addison’s disease, and menopause. This article is focused primarily on clinical studies, and work in animal models of endocrine disorders has not been included in the review.

## 2. CoQ10 and Thyroid Function

The two most common disorders of the thyroid are hyperthyroidism and hypothyroidism, resulting from over or under activity of the thyroid, respectively. Hyperthyroidism may occur for a number of reasons, but Graves’ disease is the most common. Graves’ disease is a form of hyperthyroidism characterised by overproduction of thyroid hormones, resulting from autoimmune damage to the thyroid; autoantibodies directed against the thyrotropin receptor bind to and activate the receptor, causing the autonomous production of thyroid hormones [2]. Hypothyroidism may also occur for a variety of reasons, the most common being Hashimoto’s disease, a form of hypothyroidism resulting again from autoimmune damage to the thyroid (in this case, autoantibodies directed against the thyroid antigens thyroid peroxidase and thyroglobulin) [3].

With regard to mitochondrial function, thyroid hormones regulate cellular energy metabolism via their effects on mitochondrial function [4,5]. Mitochondria are major sites of triiodothyronine accumulation within cells, where it exerts a direct effect on mitochondrial activity and energy metabolism [6]. Mitochondria are a major source of free radical production within cells [7], and the accelerating effect of triiodothyronine on basal metabolism results in an increased production of free radicals. The hypermetabolic state present in hyperthyroidism results in excessive free-radical-induced oxidative stress within cells; in contrast, the hypometabolic state induced by hypothyroidism leads to a decrease in free radical production [8,9,10].

In patients with hyperthyroidism, clinical studies have reported significantly reduced levels of CoQ10 in blood [11,12,13,14,15,16,17,18], and in thyroid tissue [19]. Ogura et al. [11] reported a mean serum CoQ10 value of 0.37 ± 0.17 µg/mL in a series of 16 patients with hyperthyroidism, significantly lower than the corresponding value of 1.01 µg/mL in normal control subjects. Similarly in a cohort of 20 hyperthyroid patients, the mean serum CoQ10 level of 0.28 ± 0.03 µg/mL was significantly lower than the value in normal controls of 0.65 ± 0.08 µg/mL [12]. In the latter study, 12 of the hyperthyroid patients were supplemented with CoQ10 (120 mg/day for 1 week); the mean serum CoQ10 level was elevated to 0.66 ± 0.05 µg/mL , with a corresponding improvement in some parameters of cardiac function, namely stroke volume and systolic time intervals (pre-ejection time and left ventricular ejection time/pre-ejection time ratio).

In the study by Mancini et al. [13], a mean plasma CoQ10 value of 0.49 ± 0.03 µg/mL was reported in a set of 8 hyperthyroid patients, significantly lower than the normal plasma CoQ10 range of 0.70–1.00 µg/mL . In a study of 21 hyperthyroid patients, Grossi et al. [14] found the mean plasma CoQ10 level to be 0.27 ± 0.13 µg/mL , significantly lower than the value for normal controls of 0.80 ± 0.20 µg/mL . Pandolfi et al. [15] reported a significantly lower mean plasma CoQ10 level of 0.51 ± 0.35 µg/mL in hyperthyroid patients, compared to the corresponding control value 0.73 ± 0.16 µg/mL .

In the study by Bianchi et al. [16], plasma CoQ10 levels were determined in a series of 22 patients with hyperthyroidism; the mean CoQ10 level of 0.63 µmol/L was significantly reduced compared to that of normal controls (0.89 µmol/L). The decrease in plasma CoQ10 levels correlated with increased oxidative stress, quantified via measurement of plasma lipid peroxide levels. In the study by Jiang et al. [17], the mean plasma CoQ10 level (0.46 µg/mL ) in a series of 38 hyperthyroid patients was significantly reduced compared to the corresponding vale in normal controls (0.65 µg/mL ).

Menke et al. [18] determined plasma CoQ10 levels in 12 children with hyperthyroidism; the mean plasma level in hypothyroid patients (0.46 µmol/L) was significantly reduced compared to that for normal controls (0.74 µmol/L). In the latter study, the plasma CoQ10 level was still significantly reduced in hyperthyroid subjects when expressed relative to cholesterol (0.169 µmol/mol) compared to controls (0.210 µmol/mol), demonstrating a lipid-independent phenomenon (lipoprotein degradation may be increased in hyperthyroidism [20]. In thyroid tissue obtained by surgical resection from 8 patients with Graves’ disease, the level of CoQ10 (12.3 ± 3.4 mg/gww), was significantly reduced compared to the CoQ10 level in normal thyroid tissue (17.0 ± 3.4 mg/gww) [19].

Possible causes for the low tissue levels of CoQ10 in hyperthyroid patients include (i) decreased synthesis resulting from competition for tyrosine, which is utilised both in CoQ10 and thyroxine biosynthesis; (ii) reduced levels of CoQ10 resulting from increased oxidative stress associated with hyperthyroidism; (iii) decreased levels of lipoprotein CoQ10 carriers in blood, either from increased degradation or reduced release from the liver.

CoQ10 deficiency has been suggested as a factor in complications of hyperthyroidism, including heart failure. Pre-clinical research and a small clinical study indicate CoQ10 supplementation may help improve cardiac performance in those with hyperthyroidism [10,12,21].

In contrast to the situation in hyperthyroidism, patients with hypothyroidism may have similar circulatory levels of CoQ10, to normal subjects [11], or substantially increased levels [12,17], precluding the necessity of CoQ10 supplementation.

## 3. CoQ10 and Pancreatic Function

Diabetes is a disorder that results when the pancreas is unable to produce the hormone insulin, or when the body is unable to respond to insulin when it is produced, in both cases leading to abnormal levels of blood glucose. Of the two main types of diabetes, type I diabetes is an autoimmune disorder in which insulin secreting beta cells in the pancreas are destroyed by the immune system. Approximately 10% of diabetes cases are of type I, which tends to occur earlier in life (mean age of onset of 13 years) [22]. Type II diabetes results when the body is unable to respond to insulin, and typically occurs later in life (mean age of onset approximately 50 years) [23]. Diabetic patients have an increased risk of cardiovascular disease, renal disease and eye disorders [24]. Mitochondrial dysfunction, oxidative stress and inflammation have been implicated in the pathogenesis of both type I and type II diabetes [25,26,27], providing a rationale for the potential role of supplemental CoQ10 in mediating these disorders. Thus, supplemental CoQ10 may benefit diabetes via several mechanisms, for example, by promoting enhanced levels of cellular energy required for glucose metabolism, via its antioxidant action, or via direct modulation of the expression of genes relevant to glucose metabolism. Blood levels of CoQ10 are reportedly reduced in both type I and type II diabetes, and a number of randomised controlled trials supplementing CoQ10 have been carried out with the objective of improving glycaemic control, reducing inflammation, or reducing the risk of heart disease, diabetic neuropathy or diabetic retinopathy, as described in the following sections.

## 4. Type I Diabetes

Blood CoQ10 levels have variously been reported to be increased [28] and decreased [29,30] in patients with type I diabetes. In a randomised controlled trial comprising 34 patients with type I diabetes, supplementation with CoQ10 (100 mg/day for 3 months) had no significant benefit on glycaemic control (blood glucose level, HbA1c, insulin dose) [31]. In an open label study of 49 type I diabetes paediatric patients, supplementation with 100 mg/day CoQ10 had no significant effect on endothelial dysfunction (soluble intracellular adhesion molecule-1) or glycaemic control (blood glucose, HbA1c) [32]. In a group of 23 patients with type 1 diabetes, supplementation with CoQ10 (200 mg/day for 3 months) significantly reduced circulatory levels of the inflammatory marker human beta defensin 1, while improving natural killer cell activity [33].

## 5. Type II Diabetes

Depleted CoQ10 levels in blood (serum and platelets) have been reported in type II diabetic patients [34]. To date, there have been 16 randomised controlled trials supplementing CoQ10 (typically 100–200 mg/day for 3–6 months) in type II diabetic patients, investigating the effects on glycaemic control, oxidative stress, inflammation and endothelial function, respectively. All studies used the ubiquinone form of CoQ10, unless otherwise indicated, and all parameters measured refer to levels in blood plasma. Eleven of these studies have reported the effect of supplemental CoQ10 on glycaemic control; six of the studies reported significant improvements in blood glucose and/or HbA1c levels [35,36,37,38,39,40], while five studies reported no significant improvement in one or both of these parameters [41,42,43,44,45].

With regard to glycaemic control, in the study by Hodgson et al. [35] of 74 type II diabetics, CoQ10 supplementation (200 mg/day for 3 months) resulted in significant reductions in HbA1c levels, as well as systolic and diastolic blood pressure. In the study by Playford et al. [36] of 80 dislipidaemic type II diabetics, supplementation with CoQ10 (200 mg/day for 3 months) significantly reduced HbA1c levels, as well as systolic blood pressure. In the study by Kolahdouz-Mohammadi et al. [37] of 64 type II diabetics, supplementation with CoQ10 (200 mg/day for 3 months) significantly reduced plasma HbA1c levels. In the study by Hosseinzadeh-Attar et al. [38] of 64 type II diabetics, CoQ10 supplementation (200 mg/day for 12 weeks) resulted in significant reduction in HbA1c and asymmetric dimethylarginine (a marker of impaired endothelial function). In the study by Mehrdadi et al. [39] of 64 overweight or obese type II diabetic patients, supplementation with CoQ10 (200 mg/day for 3 months) resulted in a significant reduction in HbA1c level, as well as weight and waist circumference. In the study by Yen et al. [40] of 50 type II diabetics, CoQ10 supplementation in the form of ubiquinol (100 mg/day for 3 months) resulted in significantly reduced HbA1c levels, together with increased levels of antioxidant enzymes (catalase, glutathione peroxidase). Yoo and Yum [41] (2018) suggested CoQ10 supplementation in patients with impaired glucose tolerance could slow the progression from pre-diabetes to overt type II diabetes.

In a randomised controlled study comprising 23 type II diabetics, administration of CoQ10 (200 mg/day for 3 months) had no beneficial effect on glycaemic control [42]. Supplementary CoQ10 (200 mg/day for 3 months) improved brachial artery endothelial function in a randomised controlled study of 40 dyslipidaemic patients with type II diabetes, although there was no benefit on glycaemic control [43]. In a randomised controlled study of 70 type II diabetics with diabetic neuropathy, CoQ10 supplementation (200 mg/day for 3 months) had no significant effect on fasting blood glucose or HbA1c levels, and had no benefit on neuropathic symptoms assessed via electromyography [44]. The randomised controlled trial reported by Moazen et al. [45] supplemented CoQ10 (200 mg/day for 2 months) in 52 type II diabetic patients, and found no significant reduction in the levels of fasting blood glucose, glycated haemoglobin or the inflammatory marker adiponectin, although the level of the oxidative stress marker malondialdehyde was significantly reduced.

In type II diabetics with diabetic retinopathy, supplementation with CoQ10 (400 mg/day for 6 months) improved circulatory levels of oxidative stress markers (lipid peroxidation products, total antioxidant capacity) [46], as well as mitochondrial function (mitochondrial membrane fluidity, ATP metabolism) [47]. A systematic review by Tabatabaei-Malazy [48] confirmed increased levels of oxidative stress and decreased levels of antioxidants in type II patients with diabetic retinopathy. In patients with diabetic neuropathy, supplementation with CoQ10 (200 mg/day for three months) did not significantly benefit neuropathic symptoms, but reduced inflammation and increased insulin sensitivity [44]. Patients with diabetic neuropathy supplemented with a daily dose of pregabalin (150 mg) and CoQ10 (300 mg) for 2 months experienced greater pain relief than with pregabalin alone [49]. Supplementation with CoQ10 (400 mg/day for 3 months) in patients with diabetic polyneuropathy resulted in decreased oxidative stress (determined via levels of lipid peroxidation products) and improved nerve conduction parameters [50]. In a randomised controlled trial comprising 40 subjects with diabetic nephropathy, supplementation with CoQ10 (100 mg/day for 3 months) significantly improved gene expression of peroxisome proliferator-activated receptor-γ, interleukin-1, and tumour necrosis factor-α [51].

To date, there have been five meta-analyses relating to CoQ10 and diabetes. The meta-analysis by Suksomboon et al. [52] based on seven randomised controlled trials found CoQ10 supplementation had no beneficial effects on glycaemic control, lipid profile or blood pressure in patients with diabetes. Based on 14 selected randomised controlled trials, Moradi et al. [53] concluded that supplementary CoQ10 significantly reduced fasting blood glucose, but not fasting insulin and HbA1c. In patients with diabetic kidney disease, CoQ10 supplementation improved fasting blood glucose and HbA1c levels, but did not significantly benefit renal function markers (meta-analysis based on four randomised controlled trials) [54]. Hajiluian et al. [55] reported CoQ10 supplementation increased circulatory total antioxidant capacity and decreased the oxidative stress marker malondialdehyde. A meta-analysis of the effect of a range of supplemental nutrients (based on 119 randomised controlled trials) found supplementary CoQ10 improved fasting blood glucose and HbA1c levels [56].

The use of statins (particularly simvastatin) has been associated with an increased risk of between 10% and 40% of developing type II diabetes [57,58]; this is thought to result from statin-induced depletions of circulatory levels of CoQ10, adiponectin and glucose transporter-4 (GLUT4) protein [59]. Although CoQ10 administration has been shown to prevent the simvastatin-induced loss of GLUT4 protein levels in cell culture [60], Kuhlman et al. [61] failed to find significant changes in muscle GLUT4 levels following supplementation with CoQ10 (400 mg/day for two months) in simvastatin-treated subjects.

## 6. CoQ10 and Infertility

The inability of women to become pregnant for at least 1 year despite regular unprotected intercourse is an indicator of infertility in one or both partners. In general terms, infertility is estimated to affect 10–20% of couples, with male infertility responsible for approximately 50% of the cases.

## 7. Female Infertility

Normal mitochondrial function is essential for oocyte maturation, fertilization, and embryo development, and reduced female fertility has been linked to mitochondrial dysfunction in oocytes [62]. Mitochondrial function and energy production deteriorate with age, adversely affecting ovarian reserve, chromosome segregation, and embryo competence [63]. Mitochondrial dysfunction and oxidative stress have also been implicated as a factor in women with reduced ovarian reserve or poor ovarian response. Ma et al. [64] described a study of 65 older women undergoing in vitro fertilisation; addition of 50 uM CoQ10 to the culture medium increased oocyte maturation rates and reduced postmeiotic aneuploidies. In a study of 170 patients with poor ovarian reserve, pre-treatment with CoQ10 for two months prior to in vito fertilisation (IVF) treatment improved the ovarian response to stimulation, together with a higher fertilization rate and more high-quality embryos [65]. A small study comprising 15 older women undergoing IVF demonstrated supplementation with CoQ10 (200 mg/day for 1 month) increased the antioxidant capacity of follicular fluid and improved oocyte quality [66].

## 8. Polycystic Ovary Syndrome

Polycystic ovary syndrome (PCOS) is the most common endocrine disorder in women of reproductive age, with a prevalence of up to 10%. PCOS is characterized by hirsutism, anovulation, hyperandrogenism and polycystic ovaries. PCOS is the cause of up to 30% of infertility in couples seeking treatment. PCOS is associated with a number of co-morbidities, including infertility, obstetrical complications, type 2 diabetes, and cardiovascular disease. The treatment of patients with PCOS has focused on relief of symptoms. The aetiology of PCOS is not completely understood, although it is thought to result from both environmental and genetic factors [67]. At the cellular level, mitochondrial dysfunction, oxidative stress and inflammation have been implicated in the pathogenesis of PCOS [68,69,70], hence the rationale for investigating the potential therapeutic role of CoQ10 in the management of PCOS.

There have been several randomised controlled clinical trials supplementing CoQ10, either alone or in combination with other substances, in PCOS. In a study comprising 86 PCOS patients, supplementation with CoQ10 over a 2-month period resulted in improved glucose homeostasis and reduced serum testosterone levels [71]. In a study with 43 overweight or obese PCOS patients, CoQ10 supplementation (200 mg/day for 2 months) resulted in a significant reduction in markers of inflammation (hs-CRP, TNFalpha, IL-6) and endothelial dysfunction (V-CAM, I-Cam, e-selectin) [72]. In a study comprising 55 PCOS patients, supplementation with CoQ10 (100 mg/day for 3 months) resulted in significant decreases in the levels of testosterone and the inflammatory marker hs-CRP [73]. Supplementation with CoQ10 (100 mg/day for 3 months) in 40 patients with PCOS resulted in significantly downregulated expression of genes associated with inflammation, including those for interleukin-1, interleukin-8 and tumour necrosis factor alpha in peripheral blood mononuclear cells [74]. In a study of 60 patients with PCOS, Samimi et al. [75] reported supplementation with CoQ10 (100 mg/day for 12 weeks) significantly reduced blood glucose, insulin and cholesterol levels compared to normal controls. In a study of 100 PCOS patients, supplementation with CoQ10 in combination with clomiphene citrate resulted in improved ovulation and pregnancy rates compared to clomiphene citrate alone [76]. The efficacy and safety of supplemental CoQ10 in PCOS has been confirmed by meta-analysis; in the meta-analysis by Zhang et al. [77], the authors concluded that “CoQ10 is a safe therapy to improve PCOS by improving insulin resistance, increasing sex hormone levels, and improving blood lipids”.

## 9. Male Infertility

The aetiology of male infertility is not completely understood, but the effect of mitochondrial dysfunction and oxidative stress on seminal fluid quality has been implicated [78,79], hence the rationale for a potential role of CoQ10 in the treatment of this disorder. Several studies have reported significantly reduced levels of CoQ10 in semen (seminal plasma and/or spermatozoa) from patients with asthenozoospermia [80,81,82].

To date, there have been 10 randomised controlled trial supplementing CoQ10 (alone or in combination) in infertile men (these studies used the ubiquinone form of CoQ10 unless otherwise indicated). In a study comprising 212 infertile men with idiopathic oligoasthenoteratospermia, supplementation with CoQ10 (300 mg/day for 26 weeks) resulted in significant improvements in sperm morphology, sperm density and sperm motility [83]. The same research group also reported improvements in the latter parameters following supplementation with the ubiquinol form of CoQ10 (200 mg/day for 6 months) in a set of 228 patients with idiopathic oligoasthenoteratospermia [84]. Balercia et al. [85] reported improved sperm motility in a cohort of 60 patients following supplementation with CoQ10 (200 mg/day for 6 months). In a study of 60 infertile men with idiopathic oligoasthenoteratozoospermia, Nadjarzadeh et al. [86] found improved sperm morphology following CoQ10 supplementation (200 mg/day for 3 months). Five randomised controlled trials supplemented CoQ10 in combination with other substances. Tang et al. [87] reported increased sperm concentration, sperm motility and sperm morphology in a study of 180 patients with idiopathic oligoasthenospermia, following supplementation with CoQ10 and tamoxifen. In a study of 260 infertile men with idiopathic oligoasthenozoospermia, administration of CoQ10 (60 mg/day) in combination with L-carnitine for 3 months resulted in significantly improved sperm concentration and sperm motility, together with an increased pregnancy rate; the improvement in these parameters was greater for CoQ10 and L-carnitine in combination, than for CoQ10 alone [88]. Supplementation with a combination of CoQ10 (200 mg/day) and selenium (200 mcg/day) for 3 months in 70 patients with idiopathic oligoasthenoteratospermia resulted in a significant improvement in sperm concentration and motility [89]. Ma and Sun [90] reported supplementation with CoQ10 (30 mg/day) and vitamin E (300 mg/day) for 3 months in a cohort of 140 infertile men with asthenozoospermia resulted in significantly improved sperm concentration and motility. Finally in a study of 80 infertile men, administration of a multi- component supplement, comprising CoQ10 plus L-carnitine, L-arginine, glutathione, zinc, vitamin B9, vitamin B12 and selenium, resulted in significantly improved sperm concentration, sperm motility and pregnancy rates [91]. Details about these randomized controlled trials are outlined in Table 1.

The efficacy of supplemental CoQ10 for the treatment of male infertility has been confirmed by three meta-analyses [92,93,94]. All three meta-analyses concluded CoQ10 supplementation resulted in improved sperm concentration, morphology and motility.

## 10. CoQ10 and Menopause

There is some evidence that hormone replacement therapy reduces blood levels of CoQ10, thereby potentially increasing the risk of cardiovascular disease in post-menopausal women [95]. Oral contraceptive use also reduces blood CoQ10 levels [96]. Nitrogen bisphosphonate drugs used to treat bone fragility disorders in post-menopausal women have also been shown to reduce blood CoQ10 levels, possibly resulting in some nitrogen bisphosphonate-associated adverse effects [97].

## 11. CoQ10, Pituitary, Adrenal and Pineal Function

With regard to disorders of the pituitary and adrenals, in patients with Cushing’s syndrome, there is evidence for mitochondrial dysfunction (assessed via respiratory chain complex enzyme activities) [98], and oxidative stress (assessed via plasma 15-F2t-Isoprostane and total antioxidant capacity) [99]. However, the role of CoQ10 in pituitary and adrenal function is an area of endocrine medicine that has been subject to relatively little research. Preliminary studies have suggested depleted circulatory levels of CoQ10 in some pituitary/adrenal disorders. Mancini et al. [100] measured plasma CoQ10 levels in a series of patients with disorders of the pituitary–adrenal axis, including 6 patients with ACTH-dependent adrenal hyperplasia; 19 patients with secondary isolated hypoadrenalism, and 19 patients with associated hypothyroidism (multiple pituitary deficiencies)**.** CoQ10 levels were significantly lower in secondary isolated hypoadrenalism than ACTH-dependent adrenal hyperplasia and multiple pituitary deficiencies. Plasma CoQ10 levels are reportedly reduced in patients with acromegaly [101].

There have been no published clinical studies in the peer-reviewed medical literature relating to the role of CoQ10 in the function of the pineal gland. With regard to the potential influence of the pineal gland on CoQ10 metabolism, in healthy adults, Niklowitz et al. [102] found daytime CoQ10 levels to be maintained within narrow limits, with a fall in levels during the night; a diurnal change in hepatic HMG-CoA reductase activity may suggest a common diurnal regulation of synthesis of both CoQ10 and cholesterol.

## 12. Determination of CoQ10 Levels

In the above sections of this article, the measurement of CoQ10 in various disorders has been described. The determination of endogenous CoQ10 status is generally based on plasma measurements, with an established reference range of between 0.5 to 1.7 µM [103]. However, plasma CoQ10 status is influenced by both diet and circulatory lipoprotein levels [103]. In view of the long circulatory half-life (approx. 24 h) of CoQ10, dietary intake may contribute up to 25% of the total amount of plasma CoQ10 [104]. The level of CoQ10 in plasma is very dependent upon the concentration of lipoproteins, which are the major carriers of CoQ10 in the circulation, with approximately 58% of total plasma CoQ10 being associated with the LDL fraction [103]. Therefore, in view of the influence of both dietary intake and lipoprotein concentration, plasma CoQ10 status may not accurately reflect cellular levels [103]. It has been suggested that plasma CoQ10 status should be expressed as a ratio relative to either total circulatory plasma cholesterol or LDL cholesterol, in order to take into account the lipoprotein status of the blood [103]. Plasma CoQ10 status is generally assessed in order to establish evidence of an underlying CoQ10 deficiency, or to monitor the efficacy of supplementation. However, at present there is no overall consensus on the appropriate level of plasma CoQ10 required to elicit therapeutic benefit to patients. In view of the above limitations, the assessment of blood mononuclear cell (MNC) CoQ10 status has been suggested as an alternative surrogate to determine endogenous CoQ10 levels in patients [103]. The level of CoQ10 in MNCs has been reported to correlate with that of skeletal muscle providing further support for the use of MNCs as an appropriate surrogate to determine endogenous CoQ10 status [105].

## 13. Summary

The three areas of endocrine medicine most investigated with regard to CoQ10 are hyperthyroidism, type II diabetes and male infertility, respectively. Clinical studies in hyperthyroid patients have consistently demonstrated substantially reduced circulatory levels of CoQ10, with some of the lowest levels recorded in human subjects. Pre-clinical research and a small clinical study indicate CoQ10 supplementation may help improve cardiac performance in those with hyperthyroidism. The outcome of studies supplementing CoQ10 in type II diabetes have been variable. Some randomised controlled trials (and meta-analyses) have reported significant benefit with regard to glycaemic control, while others have found no significant benefit; however, most studies have reported significant benefit on other diabetic parameters, such as oxidative stress, inflammation or endothelial function. In contrast to the above, randomised controlled trials (and meta-analyses) have consistently demonstrated the significant benefit of CoQ10 supplementation in male infertility, with regard to sperm morphology, sperm density and sperm motility, and increased pregnancy rates. In summary, there is arguably sufficient documented evidence to support a marketing authorisation application for this indication.

## Figures and Tables

**Table 1:** Randomised controlled trials of CoQ10 Supplementation in male infertility.

Study Reference	CoQ10 Dose/Duration	Sample Size	Study Outcome
Safarinejad [83]	300 mg/day for 26 weeks	212	Improved sperm morphology, density, and motility
Safarinejad [84]	200 mg/day for 26 weeks (ubiquinol)	228	Improved sperm density, motility, and morphology
Balercia [85]	200 mg/day for six months	60	Improved sperm parameters
Nadjarzadeh [86]	200 mg/day for 12 weeks	47	Alleviated oxidative stress but did not have significant effects on sperm parameters
Tang [87]	Coenzyme Q10 plus tamoxifen for six months	183	Increased sperm concentration, motility, and morphology
Cheng [88]	20 mg CoQ10 tid plus 10 mL L-carnitine bid for three months	262	Improved semen parameters and improved clinical pregnancy outcomes
Alahmar [89]	200 mg/day CoQ10 or 200 mcg/day selenium for three months	70	Improved sperm concentration and motility and improved antioxidant status with CoQ10 treatment
Ma [90]	30 mg/day CoQ10 plus 300 mg/day vitamin E for 3 months	140	Improved sperm concentration and motility
Kopets [91]	Multi-component supplement containing l-carnitine/acetyl-l-carnitine, l-arginine, glutathione, coenzyme Q10, zinc, vitamin B9, vitamin B12, and selenium once daily for six months	83	Improved sperm concentration and motility and improved pregnancy rates

## Data Availability

The data discussed in the review paper are available in the cited articles.
